# Involvement of *CsERF2* in leaf variegation of *Cymbidium sinense* ‘*Dharma*’

**DOI:** 10.1007/s00425-020-03426-x

**Published:** 2020-07-28

**Authors:** Jie Gao, Di Liang, Qingquan Xu, Fengxi Yang, Genfa Zhu

**Affiliations:** Guangdong Key Laboratory of Ornamental Plant Germplasm Innovation and Utilization, Environmental Horticulture Research Institute, Guangdong Academy of Agricultural Sciences, Guangzhou, 510640 People’s Republic of China

**Keywords:** Chlorophyll degradation, Chloroplast, *CsERF2*, Leaf color mutant, Transcriptome

## Abstract

**Main conclusion:**

CsERF2, an ethylene response factor, plays a role in leaf variegation.

**Abstract:**

Leaf variegation is a main ornamental characteristic in *Cymbidium sinense* (*C*. *sinense*). However, the mechanisms of leaf color variegation remain largely unclear. In the present study, we analyzed the cytological and physiological features, as well as molecular analyses of leaves from wild-type (WT) and leaf variegation mutants of *Cymbidium sinense* ‘*Dharma*’. Chloroplasts with typical and functional structures were discovered in WT and green sectors of the mutants leaves (MG), but not in yellow sectors of the mutant leaves (MY). The activities of key enzymes involved in chlorophyll (Chl) degradation and their substrate contents were significantly increased in MY. Genes related to Chl degradation also showed a significant up-regulation in MY. Transcriptomic analysis showed that the expression of all identified ethylene response factors (ERFs) was significantly up-regulated, and the 1-aminocyclopropane-1-carboxylic acid (ACC) content in MY was significantly higher compared with MG. QRT-PCR analysis validated that the expression levels of genes related to Chl degradation could be positively affected by ethylene (ETH) treatment. Stable overexpression of *CsERF2* in *Nicotiana tabacum* (*N. tabacum*) led to a decrease in Chl content and abnormal chloroplast. Transcriptomic analysis and qRT-PCR results showed that the KEGG pathway related to chloroplast development and function showed significant change in transgenic *N. tabacum*. Therefore, the leaf color formation of *C*. *sinense* was greatly affected by chloroplast development and Chl metabolism. *CsERF2* played an important role in leaf variegation of *C*. *sinense*.

**Electronic supplementary material:**

The online version of this article (10.1007/s00425-020-03426-x) contains supplementary material, which is available to authorized users.

## Introduction

*Cymbidium* is known as one of the four gentlemen (plum of blossom, *Cymbidium*, bamboo, and chrysanthemum) in Chinese classic literature. As a famous traditional flower in China, the fame of *C*. *sinense* has evolved from aesthetic value to symbolic meaning. Very early on, horticulturalists found that *C*. *sinense* has a variety of leaf color variegation, such as transparent leaves, spotted leaves, striped leaves, and yellow leaves, and they called this phenomenon ‘leaf art.’ Compared with green leaves, ‘leaf art’ varieties attract more attention and possess higher economic value (Zhang et al. 2013; Zhu et al. 2015). Even though the cultivation and breeding history of ‘leaf art’ *Cymbidium sinense* is long, it is still challenging to study the mechanism of leaf color formation due to the complicated genetic background and a lack of genomic information (Fukai et al. 2002).

Chl degradation is the immediate reason for the formation of ‘leaf art’ in ornamental plants (Li et al. 2018). The first Chl breakdown intermediate has been discovered in the early 1990s (Kräutler et al. 1991), and since then, many scientists have identified catabolic enzymes and their substrates related to Chl breakdown. The first step in Chl degradation is the conversion of chlorophyll b (Chl b) to chlorophyll a (Chl a) by Chl b reductase (CBR) (Tanaka and Tanaka 2011). Subsequently, Chla is catabolized to pheophorbide a by two types of enzymes, chlorophyllase (CLH) and pheophytin pheophorbide hydrolase (PPH) (Christ and Hörtensteiner 2014). Pheophorbide is then converted to red chlorophyll catabolite (RCC) by the catalysis of pheophorbidea oxygenase (PAO) (Pružinská et al. 2003). RCC is then reduced to *primary* fluorescent Chl catabolite (*p*FCC) by RCC reductase (RCCR) (Hörtensteiner 2013). Most enzymes involved in Chl degradation have been isolated and characterized, they form the so-called “PAO pathway.” CBR has two isoforms in plants, NON-YELLOW COLORING1 (*NYC1*), and NYC1-LIKE (*NOL*) (Sato et al. 2008). In *Arabidopsis*, *NYC1* and *NOL* are involved in the developmental processes of seed maturation and leaf senescence, while they have no effect on the regulation of Chl a/b ratio during vegetative growth (Horie et al. 2009; Tanaka and Tanaka 2011). CLH is hydrolyzed at N- and C-terminals by proteolytic enzyme in the chloroplast membranes and involved in Chl catabolism in citrus (Harpaz-Saad et al. 2007; Azoulay-Shemer et al. 2011). However, *Arabidopsis* CLHs is seemingly non-essential in Chl degradation, because the AtCLHs are located in the cytosol of senescent cells (Schenk et al. 2007). Using an elegant approach, aserine-type hydrolase located in the chloroplast, named PPH, has been found to be associated with Chl breakdown during leaf senescence in rice and *Arabidopsis* (Morita et al. 2009; Schelbert et al. 2009; Ren et al. 2010). PAO, a Fe-dependent monooxygenase, belongs to Rieske-type iron–sulfur oxygenase family. Suppression of *PAO* is known to induce premature cell death in *Arabidopsis*, maize, rice, and tomato (Gray et al. 2002; Spassieva and Hille 2002; Pružinská et al. 2003, 2005; Tanaka et al. 2003; Tang et al. 2011). *RCCR* is firstly cloned from barley and *Arabidopsis*, and it is chloroplast-targeted. *Arabidopsis* with mutated *rccr* displays cell death lesions under light during plant development through constitutive activation of defenses under normal conditions (Mach et al. 2001; Yao and Greenberg 2006; Pruzinská et al. 2007; Wüthrich et al. 2010).

As the only gaseous hormone in plant, ETH plays a vital role in plant development, growth, and response to different stresses (Alonso and Stepanova 2004). The ERFs belong to a large family of transcription factors (TFs). They are the vital factors involved in the activation of the downstream ethylene signaling pathway under multiple conditions by binding the GCC motif in the promoters of the target genes (Maren and Sergi 2015). In rice, two ERF-like genes, *Sub1A* and *Sub1C*, showed significant up-regulation during Chl degradation in leaves under submergence (Fukao et al. 2006). Similarly, four *ERF* genes in citrus have also been found to be up-regulated during fruit peel degreening (Xie et al. 2014). Yeast one-hybrid and dual-luciferase assays show that CitERF13 and ERF17 of *Malus domestica* directly bind to the GCCGAC motif in the *PPH* promoter and enhance its activity, accelerating CHL degradation (Yin et al. 2016; Han et al. 2018). In addition to participating in Chl metabolism as TFs, ERF has been found to be involved in sugar metabolite-mediated retrograde chloroplast signaling (Vogel et al. 2014). The defined chloroplast retrograde signal is a mechanism through which any stimulus perturbing chloroplast homeostasis can activate one or more retrograde signals to regulate nuclear gene expression, and ultimately feedback to chloroplast function (Bradbeer et al. 1979). Besides, sugar signaling shows an interaction with ETH in *Arabidopsis* glucose-insensitive mutant (Zhou et al. 1998; Rolland et al. 2006).

In the present study, the photosynthetic pigments content, chloroplast structure, activity of key enzymes, and the content of Chl metabolism intermediates were examined for WT and three of *C*. *sinense* ‘*Dharma*’ leaf color mutants (M1: ‘*Dharma guanyi*,’ M2: ‘*Dharma bangaoyi*,’ and M3: ‘*Dharma baoyi*’), and the gene expressions in MG and MY of mutant leaves were also compared. In addition, the gene function of *CsERF2* on *C*. *sinense* leaf variegation was analyzed. Our data revealed that *CsERF2* had crucial functions in leaf variegation of *C*. *sinense*.

## Materials and methods

### Plant material

**Fig. 1:**
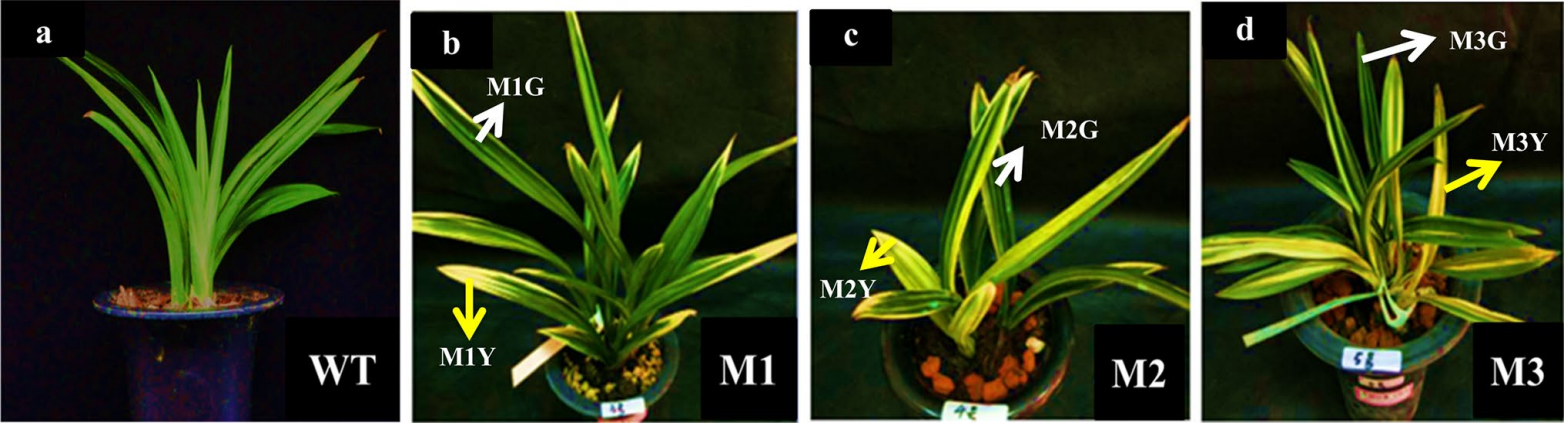
Plant phenotype of *C*. *sinense* ‘*Dharma*’ wild-type and variegated mutants. **a** WT, Green ‘*Dharma.*’ **b** M1, ‘*Dharma guanyi.*’ **c** M2, ‘*Dharma bangaoy*i.’ **d** M3,‘*Dharma baoyi.*’ White arrows denote green sectors of mutant leaves, and yellow arrows denote yellow sectors of mutant leaves

WT and three leaf color mutant (M1: ‘*Dharma guanyi*’, M2: ‘*Dharma bangaoyi*’, and M3: ‘*Dharma baoyi*’) of *C*. *sinense* ‘*Dharma*’ were selected as plant material (Fig. 1). All plants used in this study were collected from the greenhouse of Environmental Horticulture Research Institute, Guangdong Academy of Agricultural Sciences, China. All plants were maintained in pots at room temperature under a 16-h light/8-h dark photoperiod.

### Transmission electron microscopic observation

Mature green leaves from the WT and leaf color mutants of *C*. *sinense* ‘*Dharma*’ were cut into 1.0 × 1.0 mm pieces. After fixation in 2.5% glutaraldehyde at 4 °C for 4 h, the samples were rinsed with PBS thrice (10 min for each), and then fixed in 1% OsO_4_ for 2 h, followed by dehydration through a graded series of acetone. The treated material was embedded in Epon812 epoxy resin and sequentially polymerized at 37 °C, 45 °C, and 65 °C for 24 h. Ultrastructural observations were performed by a JEM-1200EX transmission electron microscope, and 70-nm thick sectors were cut by an Ultracut E ultramicrotome (Reichert-Jung; Leica, Wetzlar, Germany) and stained with 1% (w/v) uranyl acetate and 1% (w/v) lead citrate.

### Determination of key enzyme activities in Chl metabolism

Briefly, 1.0 g of leaf samples was homogenized in nine volumes of 0.01 M PBS (pH 7.4) on ice and then centrifuged at 2500*g* for 30 min. The supernatant was collected for analysis. Activity of key enzymes was determined using corresponding ELISA kits (HengYuan Biological Technology Co., Ltd., Shanghai, China). Experimental procedures were as follows: (1) Dilution of the standard: the standard was diluted according to the instructions. (2) Sample addition: blank holes (blank control holes without samples and enzyme-labeled reagents, other steps were the same) were set up, and standard holes and sample holes were also set up. Then 50 μL standard sample was accurately added to standard holes in the enzyme-coated plate. Next, 40 μL sample diluent and 10 μL sample were added to sample holes in the enzyme-coated plate. The plate was shaken gently and mixed well. (3) Incubation: the plate was sealed with sealing film and incubated at 37 °C for 30 min. (4) Liquid preparation: 30 times of concentrated washing liquid were diluted 30 times with distilled water. (5) Washing: the sealing film was carefully removed, the liquid was discarded, the plate was dried, each hole was filled with washing liquid, allowed to stand for 30 s and then discarded, and the procedure was repeated for five times. (6) Enzyme addition: 50 μL of enzyme-labeled reagent was added to each hole, except for the blank hole. (7) Incubation: the plate was sealed with sealing film and incubated at 37 °C for 30 min. (8) Washing: the sealing film was carefully removed, the liquid was discarded, the plate was dried, each hole was filled with washing liquid, allowed to stand for 30 s and then discarded, and the procedure was repeated for five times. (9) Color development: 50 μL chromogenic agent A was added, then 50 μL chromogenic agent B was added, the mixture was gently shaken, mixed well, and allowed to stand at 37 °C for 15 min in the dark. (10) Termination: 50 μL of terminating liquid was added to each hole to terminate the reaction (at this time, blue turns to yellow). (11) Measurement: the automatic microplate reader (Sunrise, Tecan, Männedorf, Switzerland) was set to zero by blank solution, and then the absorbance of each hole was measured at a wavelength of 450 nm.

### Determination of intermediates content in Chl metabolism

Briefly, 1.0 g of leaf samples was homogenized in nine volumes of 0.01 M PBS (pH 7.4) on ice and then centrifuged at 2500*g* for 30 min. The supernatant was collected for analysis. Contents of intermediates were determined using corresponding ELISA kits (HengYuan Biological Technology). Experimental procedures were as follows: (1) The required ELISA plates were taken from the aluminum foil bag after balancing at room temperature for 20 min. (2) Standard holes and sample holes were set up, and 50 μL standard solution of different concentrations were added to the standard holes. Next, 50 μL samples were added into sample holes, and the blank holes were kept empty. (3) In addition to the blank hole, 100 μL of horseradish peroxidase (HRP)-labeled detection antibody was added to each hole in the standard holes and sample holes, and the plate was sealed with a sealing plate film. Then the plate was incubated at 37 °C for 30 min. (4) The sealing film was carefully removed, the liquid was discarded, the plate was dried, each hole was filled with 350 μL washing liquid, allowed to stand for 1 min and then discarded, and the procedure was repeated for five times. (5) Subsequently, 50 μL substrate A and 50 μL substrate B were added into each hole, and the plate was incubated at 37 °C for 15 min in the dark. Next, 50 μL terminator solution was added into each hole. (6) The automatic microplate reader (Sunrise) was set to zero by blank solution, and then the absorbance of each hole was measured at a wavelength of 450 nm.

### Transcriptomic analysis

Briefly, 0.5 g of MY and MG leaves were used to extracted total RNA by Trizol method (Qiagen, Beijing, China). RNA was then reversely transcribed into cDNA using HiScript^®^ III RT SuperMix (Vazyme, Nanjing, China). The double-stranded cDNA was synthesized by using A SuperScript double-stranded cDNA synthesis kit (Life Technologies). A 454 GS-FLX instrument (Roche Applied Science) was used to sequence the double-stranded cDNA. The subsequent analysis process was performed according to our previous report (Zhu et al. 2015). The criteria used to screen of DGE were as follows: (1) FRKM of MY or MG greater than 1; (2) false discovery rate less than 0.05; (3) fold change is less than 0.5 or great than 2.

### Gene expression analysis

Briefly, 0.5 g of MY and MG leaf samples were used for RNA extraction by Trizol method (Qiagen). Subsequently, a NanoDrop 2000 spectrophotometer was used to quantify 500 ng of total RNA of MY and MG (Thermo Scientific) for reverse transcription (Vazyme). QRT-PCR was conducted on a CFX96TM Real-time PCR System (Bio-Rad, Hercules, CA, USA) with ChamQ™ Universal SYBR^®^ qPCR Master Mix (Vazyme), and data were analyzed by CFX detection system software (version 3.1). In addition, CsTUBA1 was selected as a housekeeping gene. Relative expressions of target genes at the mRNA level were calculated using the 2^−△△Ct^ method (Livak and Schmittgen 2001). Fold change less than 0.5 or great than 2 was regarded as significant change. The primers used for qRT-PCR are listed in Suppl. Table S3.

### Determination of ACC content

Briefly, 1.0 g of WT, MY, and MG leaf samples were homogenized in nine volumes of 0.01 M PBS (pH 7.4) on ice and then centrifuged at 2,500 g for 30 min. The supernatant was collected for analysis. ACC content was determined by ELISA KIT (HengYuan Biological Technology Co., Ltd.).

### ETH treatment

The green leaves of WT of *C*. *sinense* ‘*Dharma*’ were soaked in 100 μM ethephon solution for 0 h, 0.5 h, 1 h, 2 h, 3 h, and 4 h. The control group was soaked in ultrapure water. After treatment, samples were immediately frozen in liquid nitrogen and stored at − 80 °C until analysis.

### Amplification of *CsERFs* sequences

According to transcriptome sequences, 100 ng first-strand cDNA was used to amplify 10 *CsERFs* full-length cDNAs by using high-fidelity thermostable DNA polymerase (Toyobo, Osaka, Japan) with gene-specific primers. Briefly, after an initial denaturation step at 94 °C for 2 min, the amplifications were carried out with 38 cycles at a melting temperature of 94 °C for 15 s, an annealing temperature of Tm-5 °C for 30 s, and an extension temperature of 68 °C for 1 min/1000 bp. The PCR products were purified and directly cloned into the pMD-18 vector (Takara, Shiga, Japan) for sequencing. All primers used were listed in Suppl. Table S3.

### *N. tabacum* transformation

Plant transformation *CsERF2* was overexpressed in *N. tabacum* plants by a leaf disc co-cultivation method (Horsch 1985). The promoter used to drive *CsERF2* overexpression was 35S. Transgenic lines were identified by end-point PCR tests (Suppl. Fig. S1).

### Determination of starch content

Briefly, 0.1 g of WT and transgenic *N. tabacum* leaf samples were mechanical homogenized after adding 5 mL 80% alcohol, the homogenate was extracted in 80 °C water bath for 20 min, then centrifuged at 5000*g* for 10 min. Discard the supernatant and add 0.5 mL distilled water into the precipitation, then gelatinize in a water bath at 95 °C for 15 min. After gelatinization and cooling it, add 0.35 mL 9.2 mol/L perchloric acid and incubate at 25 °C for 15 min. Add 0.85 mL distilled water, shake and mix well, then centrifuge with 5000*g* for 10 min. The supernatant was collected for analysis. Starch content was determined by Plant Starch Content Determination Kit (Nanjing Jiancheng Bioengineering, Nanjing, China).

### Statistical analysis

Statistical analysis was performed using Microsoft Excel 2016 and SPSS software (https://www.ibm.com/cn-zh/analytics/spss-statistics-software). The least significant difference (LSD) test was used for significant difference analysis, and *P* < 0.05 was considered to be significantly different.

## Results

### Comparison of phenotype and chloroplast ultrastructure between WT and leaf variegation mutants of *C. sinense* ‘*Dharma*’

**Fig. 2:**
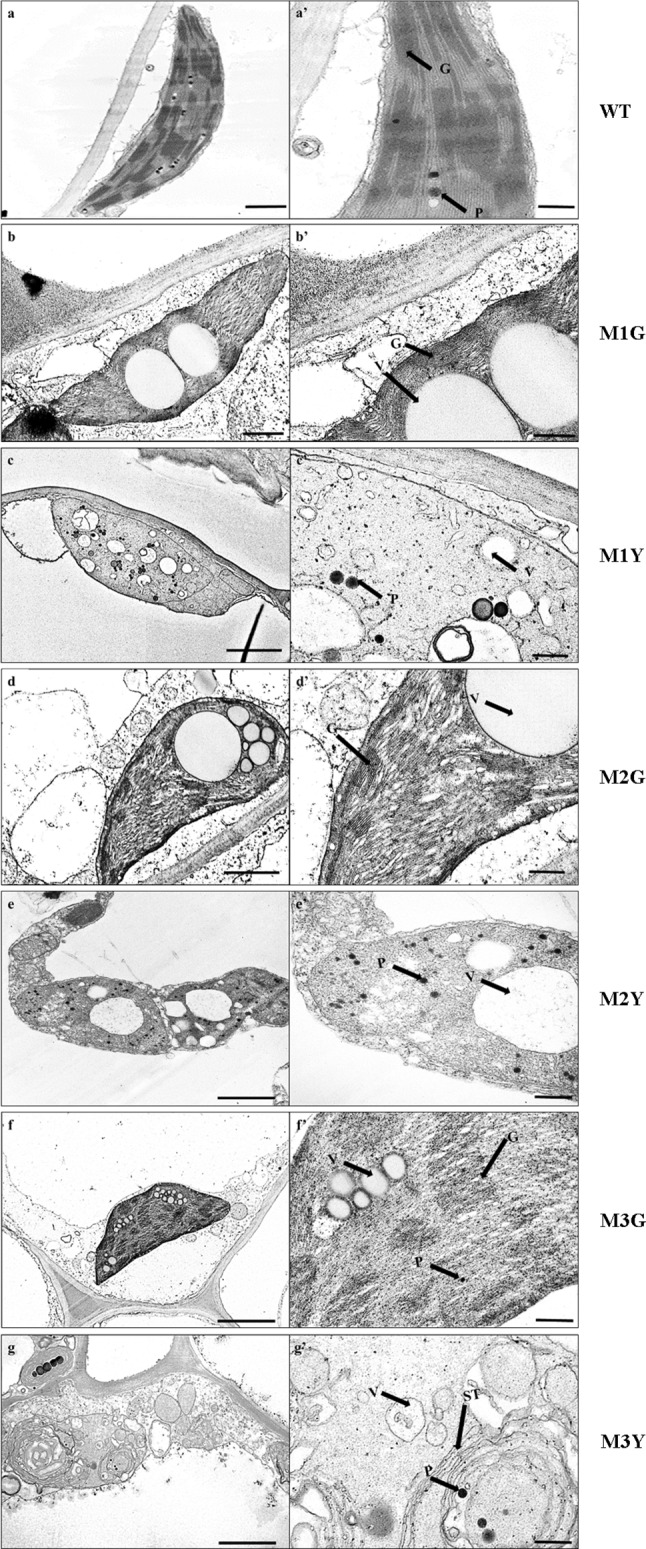
Chloroplast ultrastructure of *C*. *sinense* ‘*Dharma*’ wild-type and variegated mutants. The right panels (**aʹ**–**gʹ**) represent higher magnification of the corresponding plastid in the left panels (**a**–**g**). Chloroplast ultrastructure of **a** WT, **b** M1G, **c** M1Y, **d** M2G, **e** M2Y, **f** M3G, **g** M3Y. *G* grana, *P* plastoglobuli, *ST* stroma thylakoid, *V* vesicle-like structure. Bars in the left panels = 1 μm; in the right panels = 500 nm

WT and three of *C*. *sinense* leaf color mutants (named M1: ‘*Dharma guanyi,*’ M2: ‘*Dharma bangaoyi,*’ and M3: ‘*Dharma baoyi*’) were selected as plant materials. The leaf shape of WT and mutants was similar. Compared with green leaves of the WT plant, leaves of the mutants showed a variegated phenotype (Fig. 1). The ultrastructure of chloroplasts was observed in WT and mutants. The chloroplast ultrastructure of WT plants contained typical functional structures, including complete membrane envelope, granum thylakoids, starch grains, and plastoglobuli (Fig. 2a). The morphology of MG chloroplasts was altered compared with the WT, and the size of MG starch grains was bigger compared with WT, while the typical functional structures could still be observed (Fig. 2b, d, f). In MY, chloroplast morphology and structure showed pathological changes, and only vesicle-like structures and plastoglobuli could be observed (Fig. 2c, e, g). In summary, the chloroplast development of MY was impaired.

### Key enzymes activity and intermediates content in Chl degradation were significantly up-regulated in MY

**Fig. 3:**
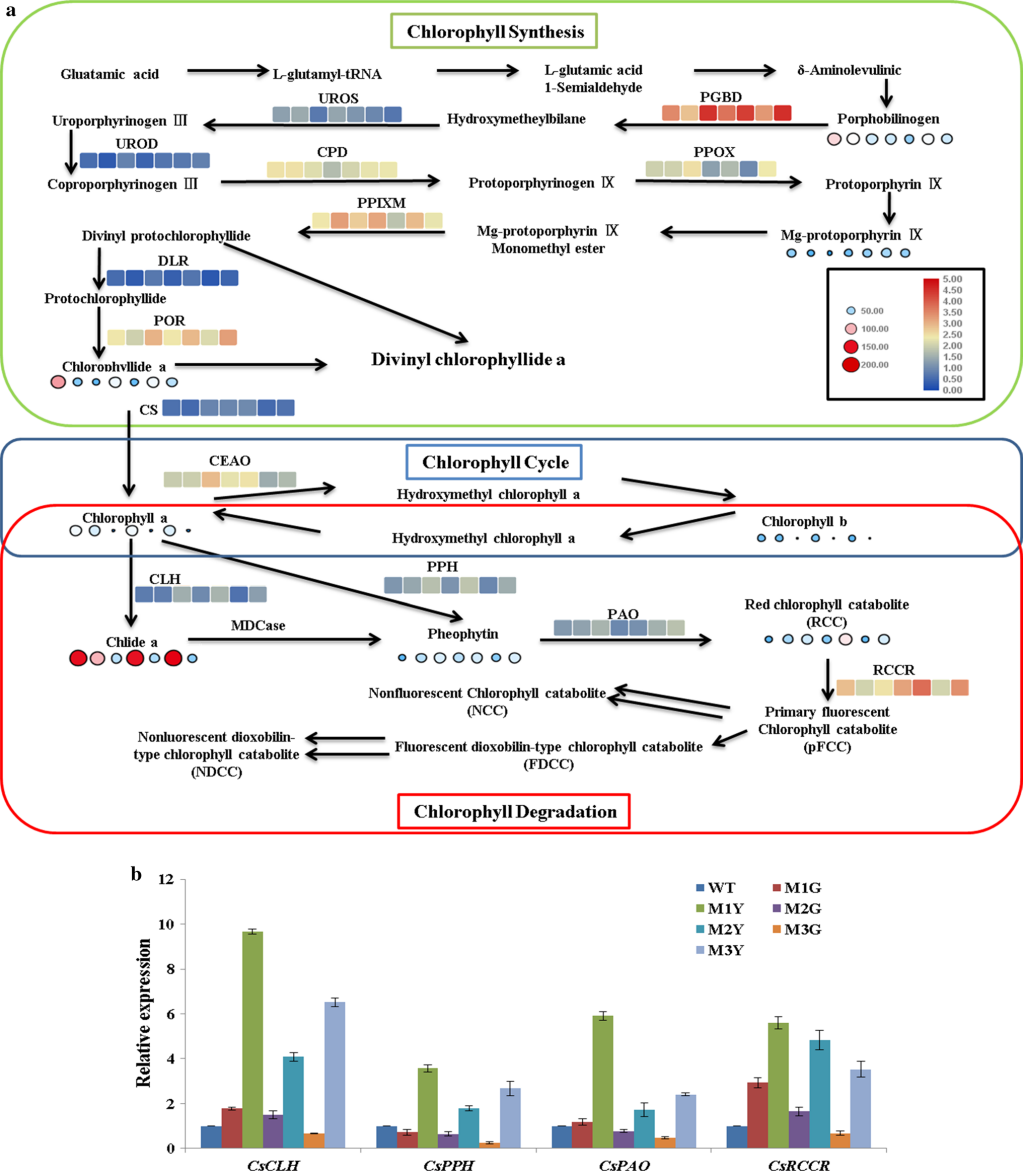
**a** The altered patterns of enzymes activity and intermediates content in WT, MG, and MY of *C*. *sinense* ‘*Dharma*’ under Chl metabolism. Boxes indicate enzyme activity, circles indicate intermediate product contents. The heat maps of genes related to Chl degradation, from left to right, show WT, M1G, M1Y, M2G, M2Y, M3G, and M3Y. **b** qRT-PCR expression analysis of *CsCLH*, *CsPPH*, *CsPAO,* and *CsRCCR* in WT, MG, and MY of *C*. *sinense* ‘*Dharma.*’ Data represent the means ± SE of at least three replicates

The photosynthetic pigments content is significantly decreased in MY in our previous research (Zhu et al. 2015), and we have speculated that the activities of key enzymes involved in Chl metabolism may also be significantly changed in MY. Activities of 15 key enzymes and content of six intermediates were determined to analyze Chl biosynthesis and degradation pattern in MY compared with WT and MG. The results were illustrated by a Chl metabolism pathway, according to the results reported by Beale (Beale 2005), Ayumi (Tanaka and Tanaka 2006), and Hortenseiner (Hortensteiner 2013) (Fig. 3a). Compared with WT and MG, the activities of all enzymes related to Chl degradation and the content of their substrates were all significantly increased in MY. We also assessed the expression of *CLH*, *PPH*, *PAO,* and *RCCR* using qRT-PCR (Fig. 3b). All four genes were significantly up-regulated in MY compared with WT and MG. Based on these results, we inferred that the Chl degradation pathway might play a critical role in leaf color variegation.

### Analysis of differentially expressed genes (DEGs)

The transcriptome was used to analyze the difference between MG and MY at the transcript level. As a result, 49,887 and 51,914 unigenes were found in MY and MG cDNA library, respectively. A total of 521 DEGs were identified, including 398 up-regulated DEGs and 123 down-regulated ones (Suppl. Table S1).

Using gene ontology (GO) annotation, 328 of 521 DEGs were classified into three different categories (molecular function, cellular component, and biological process). ATP binding (GO: 0005524), nucleic acid binding (GO: 0003676), and DNA binding (GO: 0003677) were the top three enriched GO terms in molecular function category (Suppl. Fig. S2). In the cellular component category, the top three significantly enriched GO terms were an integral component of membrane (GO: 0016021), membrane (GO: 0016020), and nucleus (GO: 0005634). Oxidation–reduction process (GO: 0055114), regulation of transcription (DNA-templated) (GO: 0006355), and protein phosphorylation (GO: 0006468) were the top three significantly enriched GO terms in biological function category.

In these DEGs, two phospholipase A1 (*PLA1*) genes (comp38295_c0 and comp27001_c0) and monogalactosyl diacylglycerol (*MGDG*) synthase gene were significantly up-regulated in MY, which had function in lipid metabolism in the chloroplast membrane. In addition, three DEGs (comp28163_c0, comp10875_c0, and comp13715_c0) involved in the photosynthetic system were significantly down-regulated in MY, namely photosynthetic NDH subunit of subcomplex B2 (PNSB2), photosystem II protein D1 (psbA), and M-type thioredoxin (Trx) (Suppl. Table S1).

### ERFs are all significantly up-regulated in MY

**Fig. 4:**
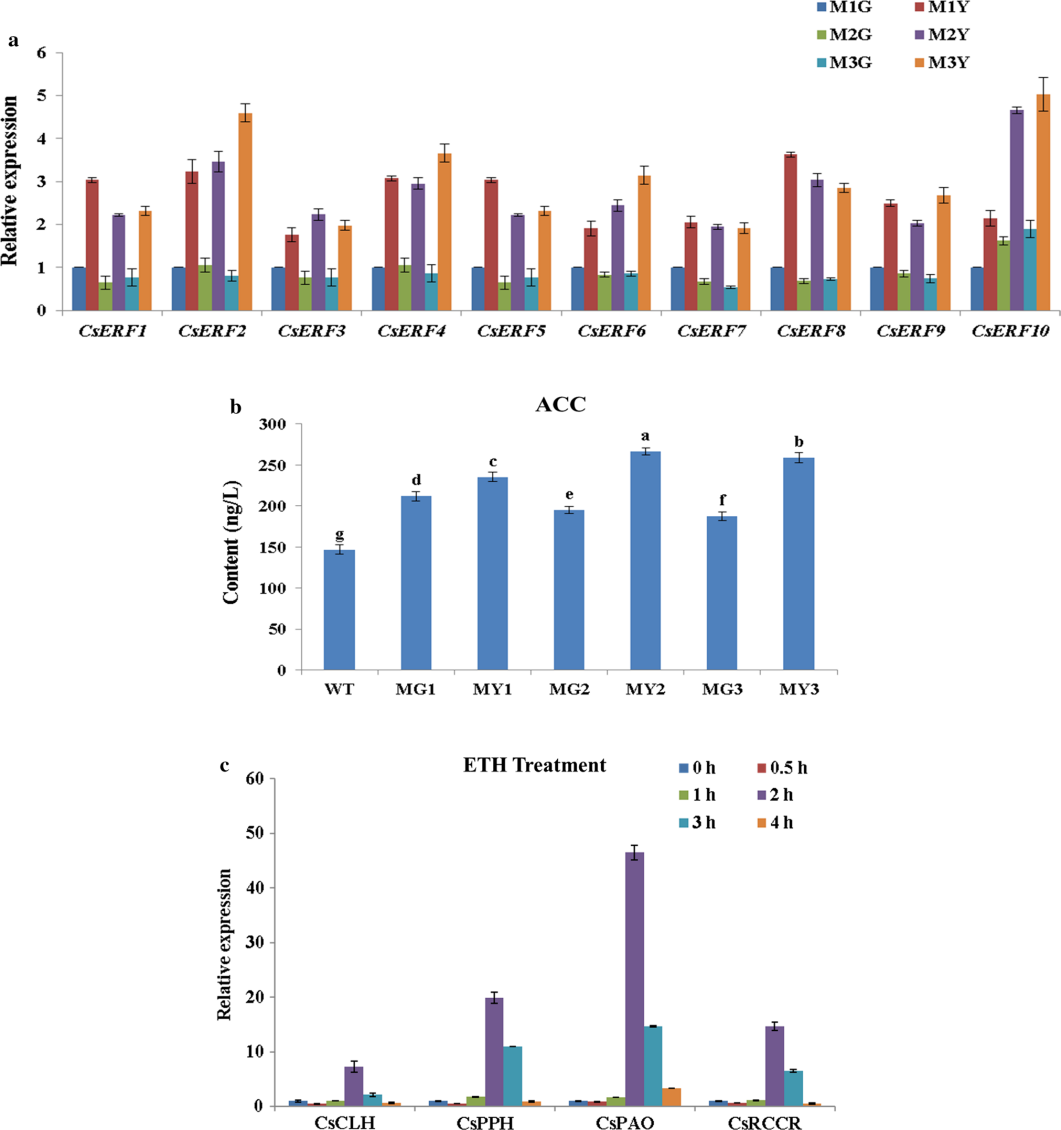

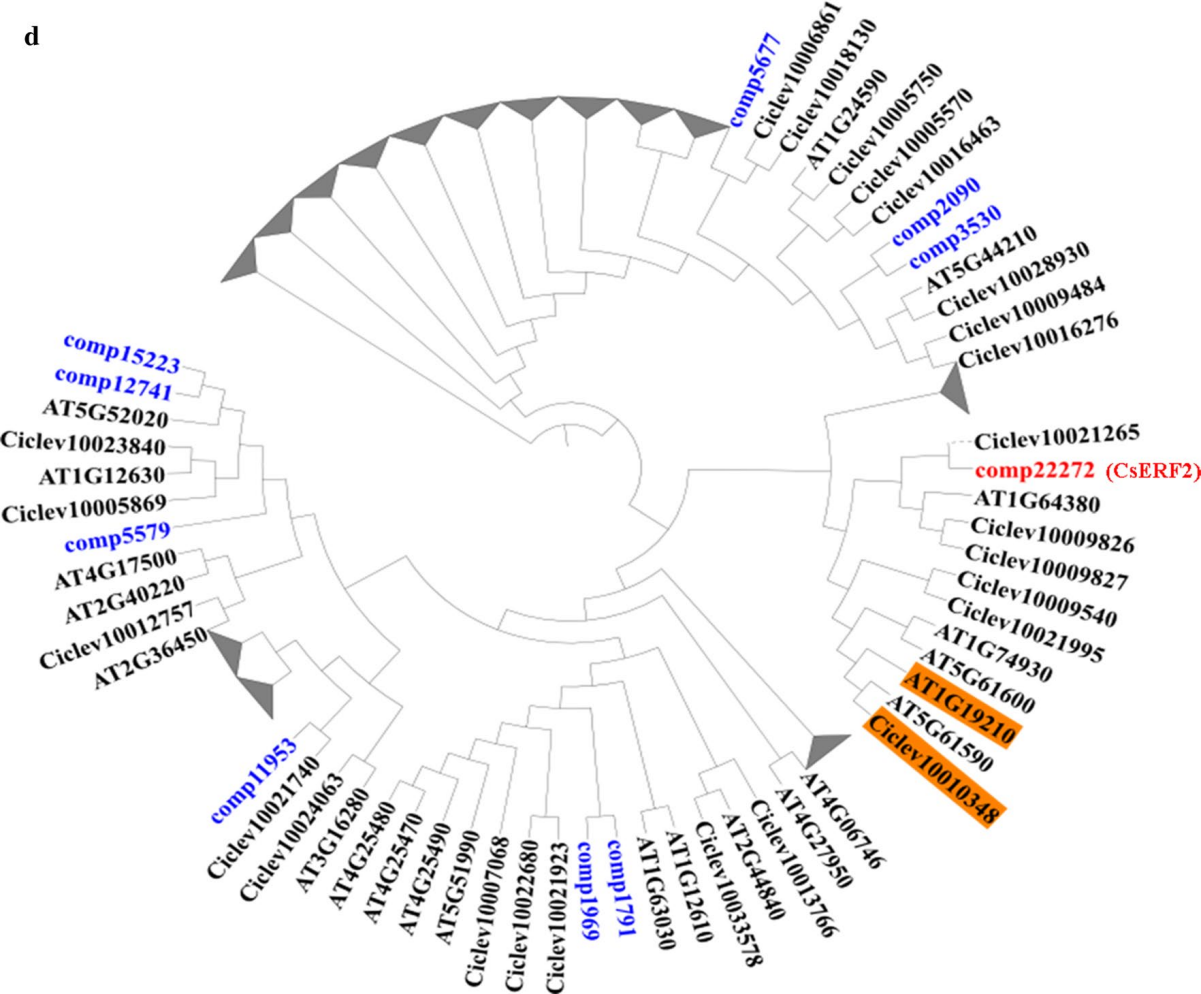
**a** qRT-PCR expression analysis of identified CsERFs between MG and MY of *C*. *sinense* ‘*Dharma.*’ **b** The ACC contents in WT, MG, and MY of *C*. *sinense* ‘*Dharma.*’ Different letters are used to indicate the means that differ significantly (*P* < 0.05). **c** qRT-PCR expression analysis of Chl degradation-related genes under ETH treatment in WT of *C*. *sinense* ‘*Dharma.*’ **d** Phylogenetic analysis of 10 CsERFs identified in transcriptome. The deduced amino acid sequences of *Arabidopsis* and citrus AtAP2/ERF genes were obtained from TAIR and citrus genome. The phylogenetic trees were constructed using Mega7. Data represent the means ± SE of at least three replicates

Based on the transcriptomic analysis, we found that the expressions of all *ERFs* identified in the transcriptome were significantly up-regulated in MY compared with MG (Suppl. Table S1). To confirm the transcriptomic data and verify the expression profiles of *ERFs*, qRT-PCR was performed to validate the expression levels of *ERFs* in MG and MY. Results indicated that the expressions of *ERFs* at the transcript level were all significantly up-regulated in yellow sectors compared with green sectors of the mutant plant (Fig. 4a). ERFs are critical downstream factors of the ethylene signaling pathway (Xie et al. 2017). Therefore, we speculated that the content of ethylene (ETH) in yellow sectors might be significantly altered. To confirm this hypothesis, the contents of ACC (substrate of ethylene) in WT, as well as mutant leaves, were determined. Compared with WT, the ACC content was elevated in both MY and MG, while the ACC content in MY was significantly compared with MG (Fig. 4b). Moreover, to test whether ETH treatment could affect the expression level of Chl degradation-related genes, WT plants were treated by ethephon for 0 h, 0.5 h, 1 h, 2 h, 3 h, and 4 h. The expression of *CsCLH*, *CsPPH*, *CsPAO,* and *CsRCCR* was all significantly up-regulated after 2 h of treatment (Fig. 4c). In general, the expression pattern of ERFs was consistent with the content of ACC, and the expression of Chl degradation-related genes could be positively regulated by ETH treatment. We, therefore, concluded that the variegation of mutant leaves could be highly affected by the regulation of ERFs.

The sequences of 10 CsERFs identified in the transcriptome were amplified by PCR with high-fidelity thermostable DNA polymerase method, and PCR products were sequenced. To identify the key ERF involved in leaf variegation, these 10 CsERFs were grouped by phylogenetic tree analysis. For the benefit of the analysis, we generated a simplified version of the phylogenetic tree (Fig. 4d). CitERF13 (Ciclev10010348) displays an important function in post-harvest degreening (Yin et al. 2016), and in our result, comp22272 (CsERF2) had the highest homology with CitERF13. Based on the above analysis, CsERF2 was selected as a key factor in leaf variegation.

### Transgenic analysis of *CsERF2* in *N. tabacum*

**Fig. 5:**
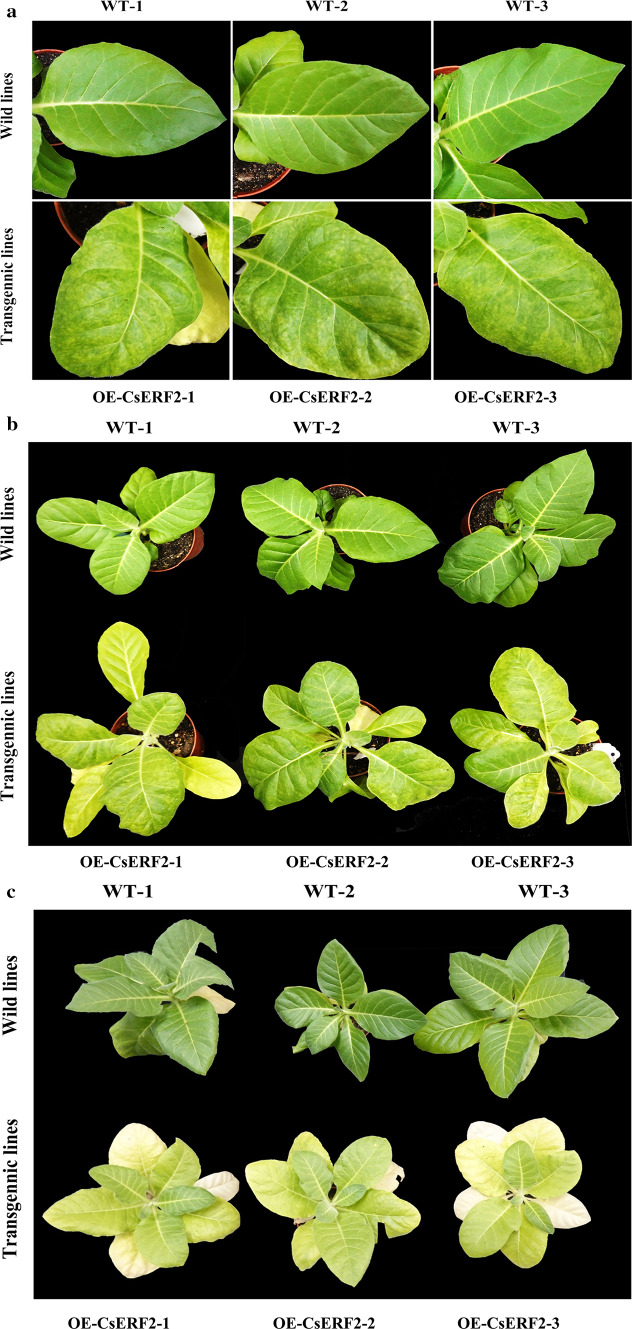
**a** Leaves phenotype comparison between wild-type and transgenic *N. tabacum* lines after 2 months of cultivation under normal condition. **b** Plants phenotype comparison between wild-type and transgenic *N. tabacum* lines after 2 months of cultivation under normal condition. **c** Plants phenotype comparison between wild-type and transgenic *N. tabacum* lines after 3 months of cultivation under normal condition. *WT* wild-type, *OE-CsERF2* transgenic *N. tabacum* lines

**Fig. 6:**
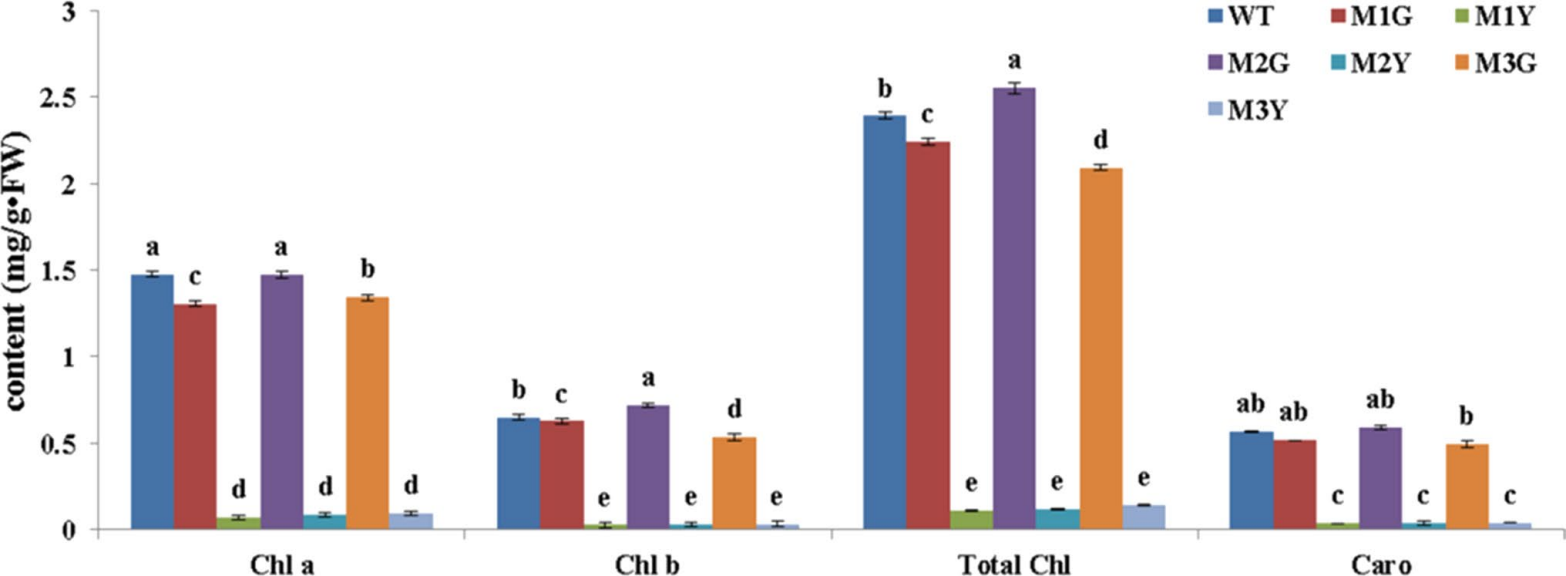
Photosynthetic pigment contents in wild-type and transgenic *N. tabacum* plants. Data represent the means ± SE of at least three replicates. Different letters are used to indicate the means that differ significantly (*P* < 0.05)

**Fig. 7:**
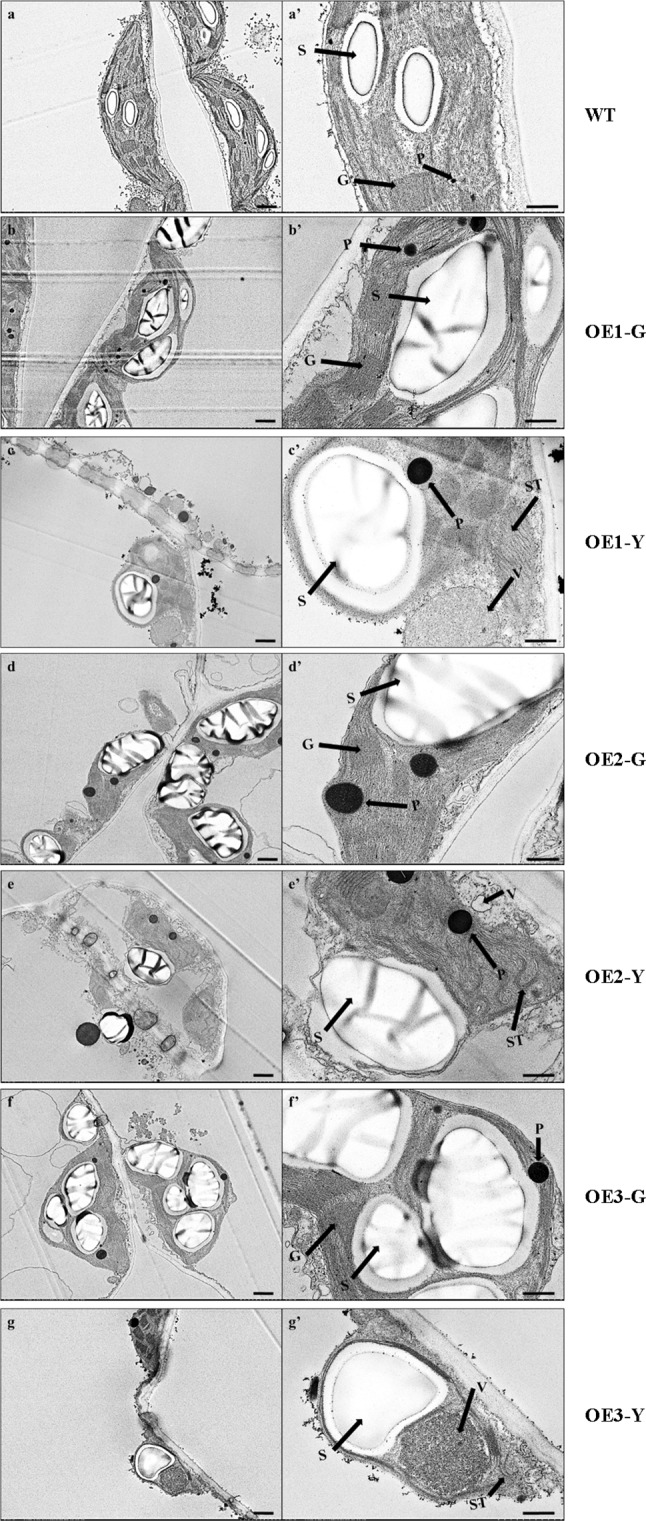
Chloroplast ultrastructure of *N. tabacum* WT and transgenic lines. The right panels (**aʹ**–**gʹ**) represent higher magnification of the corresponding plastid in the left panels (**a**–**g**). Chloroplast ultrastructure of **a** WT, **b** OE1-G, **c** OE1-Y, **d** OE2-G, **e** OE2-Y, **f** OE3-G, **g** OE3-Y. *G* grana, *P* plastoglobuli, *S* starch grain, *ST* stroma thylakoid, *V* vesicle-like structure. Bars in the left panels = 1.0 μm; in the right panels = 500 nm

**Fig. 8:**
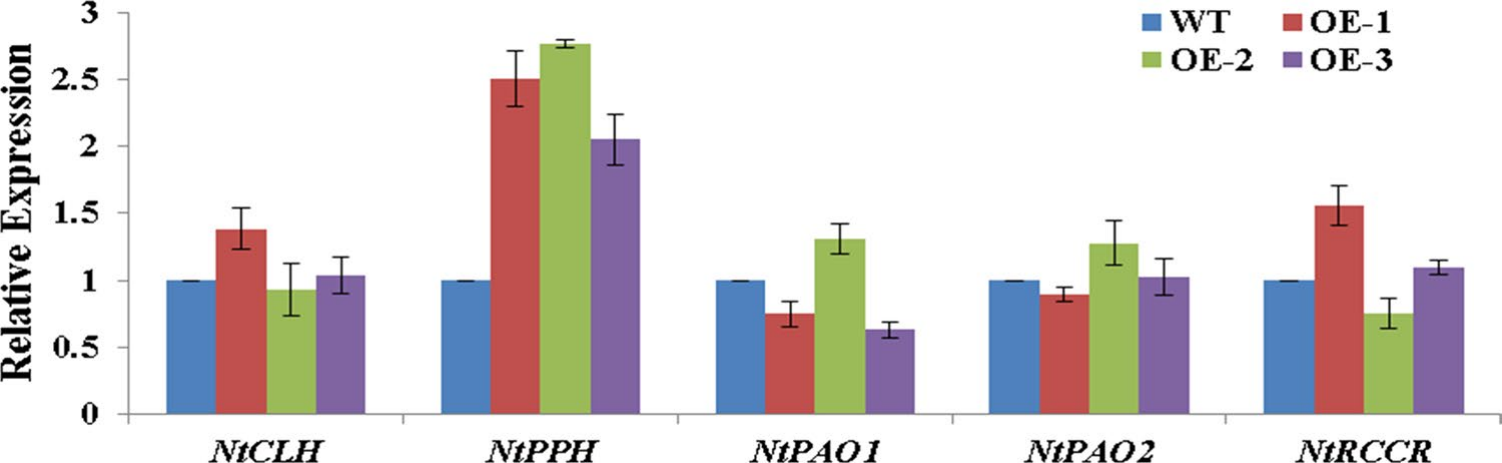
Expression of genes related to Chl degradation in transgenic *N. tabacum* lines. Relative expression was calibrated with WT. Data represent the means ± SE of at least three replicates

The function of *CsERF2* was characterized through a stable transformation in *N. tabacum*. After 2 months of cultivation under normal conditions, the leaves of *CsERF2* transgenic plants presented a mottled phenotype (Fig. 5a, b). The mottled leaves turned yellow after 3 months of cultivation, followed by the appearance of mottling on new leaves (Fig. 5c). *CsERF2* overexpression induced the loss of Chl and carotenoid content in transgenic lines (Fig. 6). Besides, chloroplast ultrastructure of transgenic lines also appeared to be significantly changed. The number of chloroplasts in yellow sectors of transgenic leaves was reduced compared with the WT and the green sectors of transgenic leaves. Structurally, chloroplasts in the green sectors of transgenic leaves were similar to WT, while the starch grains were significantly larger. The chloroplast structure was destructed in the yellow sectors of transgenic leaves, the double membranes were damaged, the grana lamellae were disorganized, and the chloroplast contained vesicles (Fig. 7). Expression levels of *N. tabacum* genes related to Chl degradation were also examined in transgenic lines. The expression level of *NtPPH* was substantially enhanced in *CsERF2*-overexpressing transgenic *N. tabacum* compared with WT (Fig. 8).

Transcriptomic analysis was used to compare the transcript state between WT and *CsERF2*-overexpressing transgenic *N. tabacum* lines. KEGG analysis showed that metabolic pathways related to chloroplast development and function (photosynthesis pathway, photosynthesis-antenna proteins pathway, starch and sucrose metabolism pathway, carbon fixation in photosynthetic organisms, porphyrin and chlorophyll metabolism pathway) were significantly changed, which indicated that the overexpression of *CsERF2* in *N. tabacum* did change the chloroplast function and induce the phenotype of leaf color variegation.

## Discussion

Leaf variegation is an important ornamental characteristic. In *C*. *sinense*, leaf variegation has high economic value. Though leaf-variegated varieties of *C*. *sinense* have been bred for hundreds of years, the regulatory mechanisms of leaf variegation remain largely unexplored because of the long juvenile phases and complicated genetic background (Fukai et al. 2002). In the present study, WT and three leaf color mutants (M1: ‘*Dharma guanyi*’, M2: ‘*Dharma bangaoyi*’, and M3: ‘*Dharma baoyi*’) in *C*. *sinense* ‘*Dharma*’ were used as research objects to understand the regulatory mechanisms in leaf color formation. We obtained a comprehensively better understanding of leaf color formation in *C*. *sinense* using cytological, physiological, and molecular biological methods.

The decreased Chl contents are often linked with the abnormal structure of the chloroplast (Li et al. 2018). Any developmental defect of the chloroplast can negatively regulate the stability of photosynthetic pigments, thus changing the content and proportion of photosynthetic pigments, ultimately leading to leaf variegation (Yang et al. 2015). In our study, the chloroplast membrane of the yellow sectors was impaired. Similarly, chloroplasts with well-organized membrane are only found in the green sectors but not in the yellow sectors of the leaf color mutants of a moth orchid, bamboo, rice, *Anthurium andraeanum,* and *Ginkgo biloba* L. (Lin et al. 2008; Gong et al. 2013; Tsai et al. 2017; Li et al. 2018). In order to decipher the reason for decreased Chl content in *C*. *sinense*, we determined the activity of key enzymes and content of intermediates in Chl synthesis and degradation (Fig. 3a). In Chl synthesis, there were no obvious differences between MY and MG in terms of the changes of enzyme activity and intermediates content, and our previous study has shown that the expression level of the key genes related to Chl biosynthesis is not different between MY and MG (Zhu et al. 2015). However, the study of gold leaf coloration in *Ginkgo biloba* L. and Burley Tobacco has revealed that the content of precursors involved in Chl synthesis is significantly decreased (Liu et al. 2015; Li et al. 2018). In the Chl degradation pathway, the activity of key enzymes (CLH, PPH, PAO, and RCCR) and content of intermediates were all significantly increased in MY, and the expression of *CsCHL*, *CsPPH*, *CsPAO*, and *CsRCCR* was also significantly up-regulated in MY (Fig. 3b). Taken together, our results suggested that the rate of Chl degradation was faster in MY compared with the WT and MG, indicating that the balance between Chl synthesis and breakdown was skewed in yellow sectors of the mutant plant.

ERF is a TF involved in fruit degreening in citrus and apple by binding to the GCC box in the PPH promoter to induce the expression level of PPH (Yin et al. 2016; Han et al. 2018). In our present study, all identified CsERFs showed significant up-regulation in MY (Fig. 4a) and the overexpression of *CsERF2* in *N. tabacum* also induced the expression of *NtPPH* (Fig. 8a). Transcriptomic analysis was used to compare the transcript state between WT and *CsERF2*-overexpressing transgenic *N. tabacum*. By bioinformatics analysis, starch and sucrose metabolism showed significant changes (16 differential genes) in transgenic *N. tabacum* compared with WT (Suppl. Fig. S3 and Suppl. Table S2), which was consistent with the dramatically increase in starch grain size and significant decrease of starch content (Suppl. Fig. S4) in transgenic *N. tabacum*. In reported studies of retrograde signals in chloroplasts, sugar-related metabolite is regarded as an important signal, and transduction crosstalk has been found in glucose and ethylene signal (Zhou et al. 1998; Rolland et al. 2006; Vogel et al. 2014). Sugar sensing, involving a *hexokinase* (*HXK*, G4097_41452 and G4097_9857) (Xiao et al. 2000; Moore et al. 2003), showed significant down-regulation in transgenic *N. tabacum*. *TP/phosphate translocator* (*TPT*, G4097_56597) which is responsible for the transport of triose phosphates (TP) (Fliege et al. 1978) was also significantly down-regulated in transgenic *N. tabacum*. Besides, *Genome Uncoupled 1* (*GUN1*, G4097_264), playing an important role in relaying the plastid signals to nucleus, was significantly up-regulated in transgenic *N. tabacum*. To further understand the change of chloroplast retrograde signaling in *CsERF2*-overexpressing transgenic *N. tabacum*, we analyzed the expression of nucleus genes associated with chloroplast retrograde signaling by RT-PCR. The qRT-PCR results showed that most detected nucleus genes related to the tetrapyrroles metabolism (Mochizuki et al. 2001; Larkin et al. 2003; Strand et al. 2003), plastid gene expression signaling pathway (Waters et al. 2009; Woodson et al. 2013; Hu et al. 2014; Leister et al. 2014), and plastid metabolism (Mou et al. 2000; Estavillo et al. 2011; Mandal et al. 2012; Xiao et al. 2012; Vogel et al. 2014) showed significant down-regulation in transgenic *N. tabacum* (Suppl. Fig. S4). The excessive accumulation of starch grains, significant decrease of starch content, and the differential expression of key nucleus genes related to chloroplast retrograde signaling in overexpressed *N*. *tabacum* suggested that one potential possibility of CsERF2 function was to regulate the expression of nucleus genes by triggering the retrograde signals in chloroplasts through sugar signals. In the next step, we will study the mechanism by which *CsERF2* regulates the sugar signals to affect the retrograde signal of chloroplast.

It is worth noting that the conclusions of this paper were made based on physiological, biochemical, and transcriptional analyses, while there were no specific results on post-transcriptional modification and post-translational changes. At present, we have begun to analyze the protein abundance and post-translational modification changes of *C*. *sinense* ‘*Dharma,*’ in order to obtain more comprehensive results.

## Conclusions

Collectively, the mechanisms of leaf variegation of *C*. *sinense* were analyzed by using the methods of TEM observation, enzymatic determination, content determination, transcriptomic analysis, qRT-PCR, and gene function identification. Our analysis on the ultrastructure of chloroplasts and physiological characteristics showed that there were distinct differences between leaf variegation mutants and WT of *C*. *sinense* ‘*Dharma.*’ The expression of all identified *ERFs* was significantly up-regulated in MY compared with MG and qRT-PCR analysis verified that the expression of genes related to Chl degradation could be positively affected by ETH treatment. Overexpression of *CsERF2* in *N. tabacum* results in leaf variegation and CsERF2 regulated the expression of genes associated with chloroplast development and function.

### *Author contribution statement*

GZ and FY conceived and designed the experiments. JG performed the experiments and wrote the paper. DL performed the experiments. QX contributed reagents/materials/analysis tools.

## Electronic supplementary material

Below is the link to the electronic supplementary material.

Supplementary file1 Suppl. Fig. S1 End-point PCR tests of transgenic N. tabacum with CsERF2 over-expression (TIF 1149 kb)

Supplementary file2 Suppl. Fig. S2 Go terms significantly enriched in the DEGs between MG and MY (TIF 819 kb)

Supplementary file3 Suppl. Fig. S3 Differential expression genes in starch and sucrose metabolism pathway between WT and CsERF2-overexpressing transgenic N. tabacum (TIF 1972 kb)

Supplementary file4 Suppl. Fig. S4 Starch content in WT and transgenic N. tabacum plants. Data represent the means ± SE of at least three replicates. Different letters are used to indicate the means that differ significantly (P<0.05) (TIF 445 kb)

Supplementary file5 Suppl. Fig. S5 Expression of genes a tetrapyrrole metabolism, b plastid gene expression signaling pathway, c plastid metabolism in transgenic N. tabacum. Relative expression was calibrated with WT. Data represent the means ± SE of at least three replicates (TIF 747 kb)

Supplementary file6 (XLSX 74 kb)

Supplementary file7 (XLSX 4900 kb)

Supplementary file8 (DOCX 16 kb)

## Abbreviations

ACC: 1-Aminocyclopropane-1-carboxylic acid
CLH: Chlorophyllase
MG (MY): Green (yellow) sectors of the mutant leaves
PPH: Pheophytin pheophorbide hydrolase
PAO: Pheophorbidea oxygenase
RCCR: Red chlorophyll catabolite reductase
ERF: Ethylene response factor
ETH: Ethylene
