# Supplementary figures and images for: Involvement of CsERF2 in leaf variegation of Cymbidium sinense ‘Dharma’
Source: Planta. 2020 Jul 28;252(2):29. doi: 10.1007/s00425-020-03426-x (PMC7387381; doi:10.1007/s00425-020-03426-x)

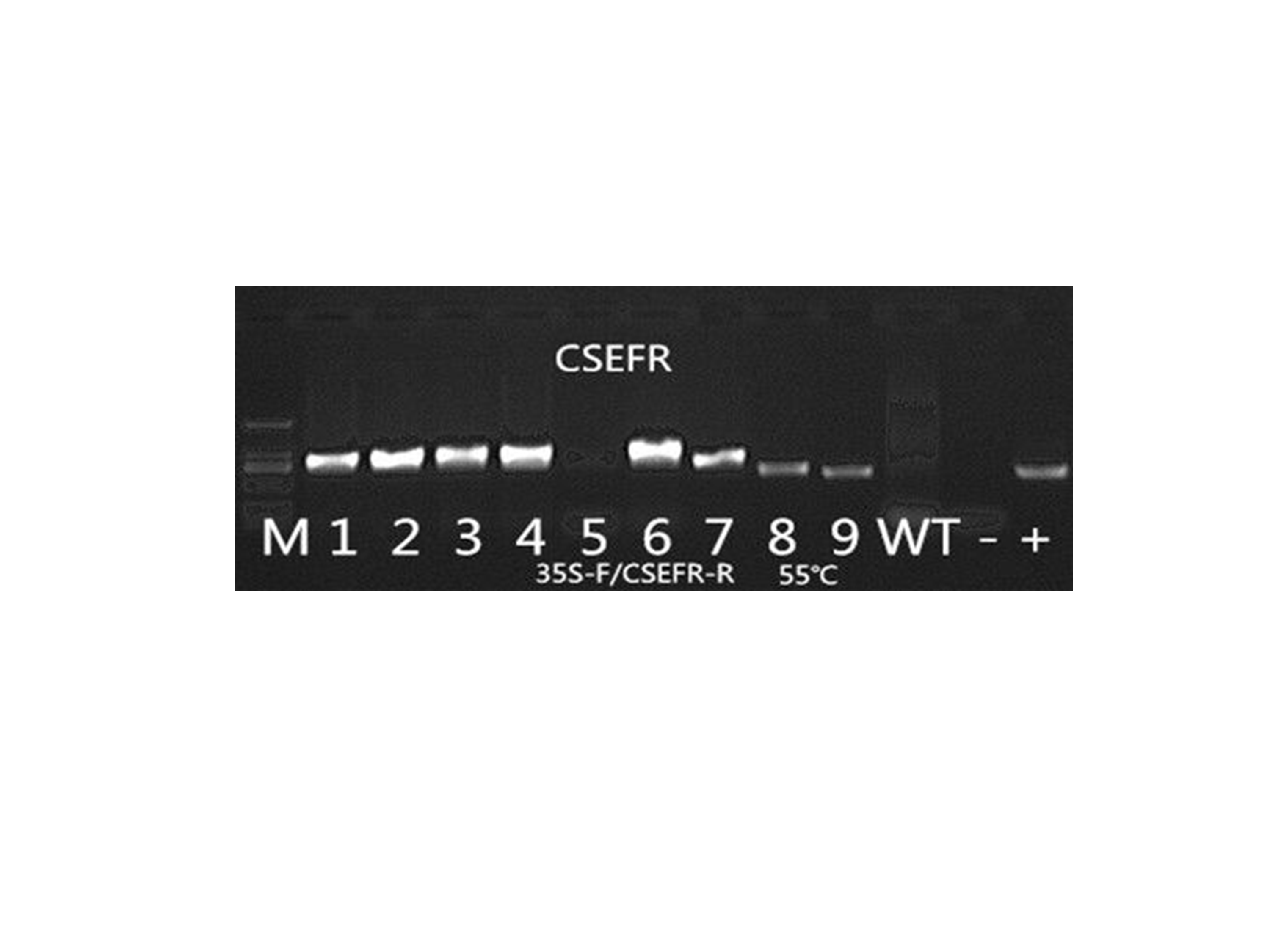

Supplement: Supplementary file 1 — Supplementary file1 Suppl. Fig. S1 End-point PCR tests of transgenic N. tabacum with CsERF2 over-expression (TIF 1149 kb) [file 425_2020_3426_MOESM1_ESM.tif]

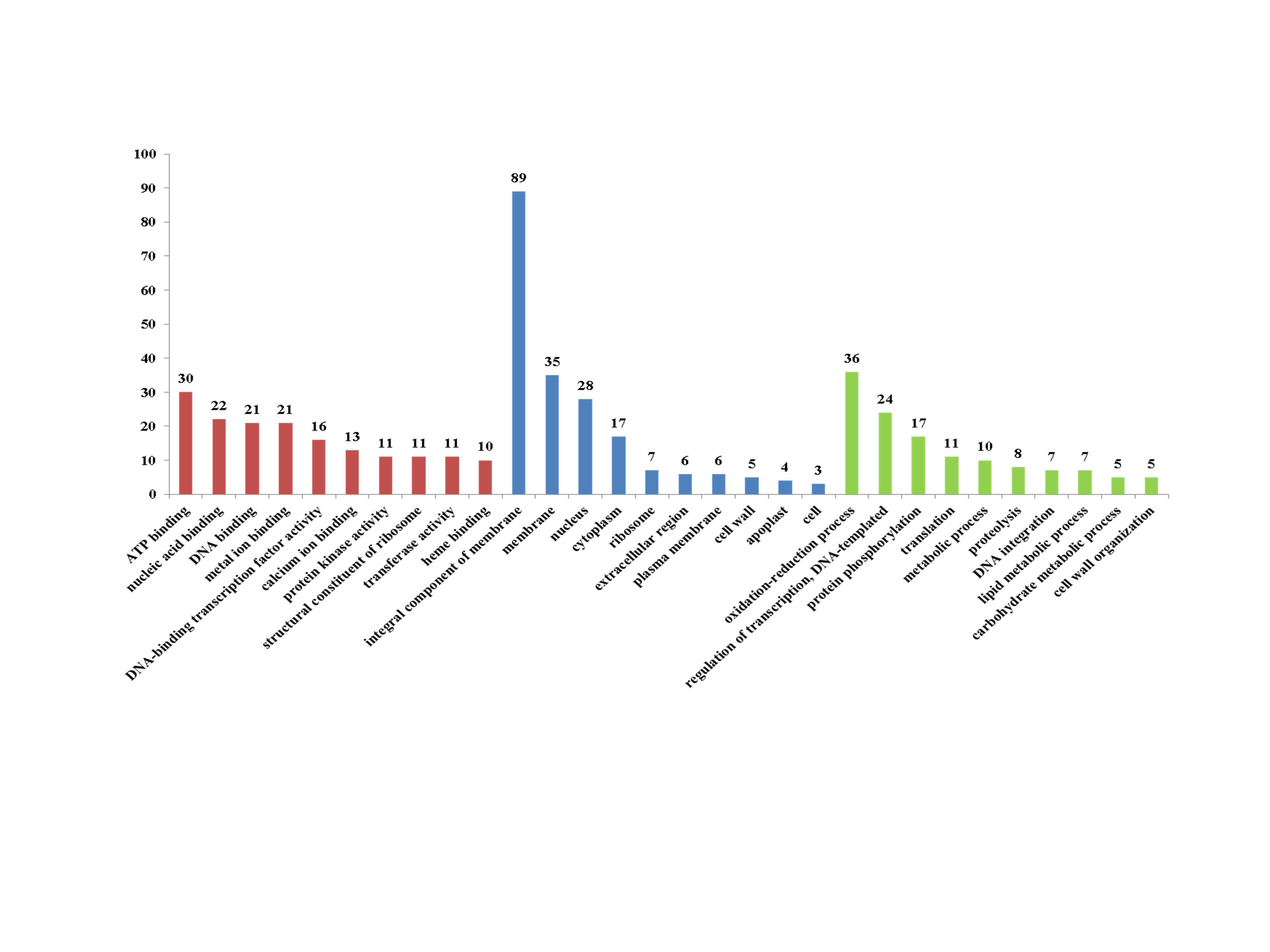

Supplement: Supplementary file 2 — Supplementary file2 Suppl. Fig. S2 Go terms significantly enriched in the DEGs between MG and MY (TIF 819 kb) [file 425_2020_3426_MOESM2_ESM.tif]

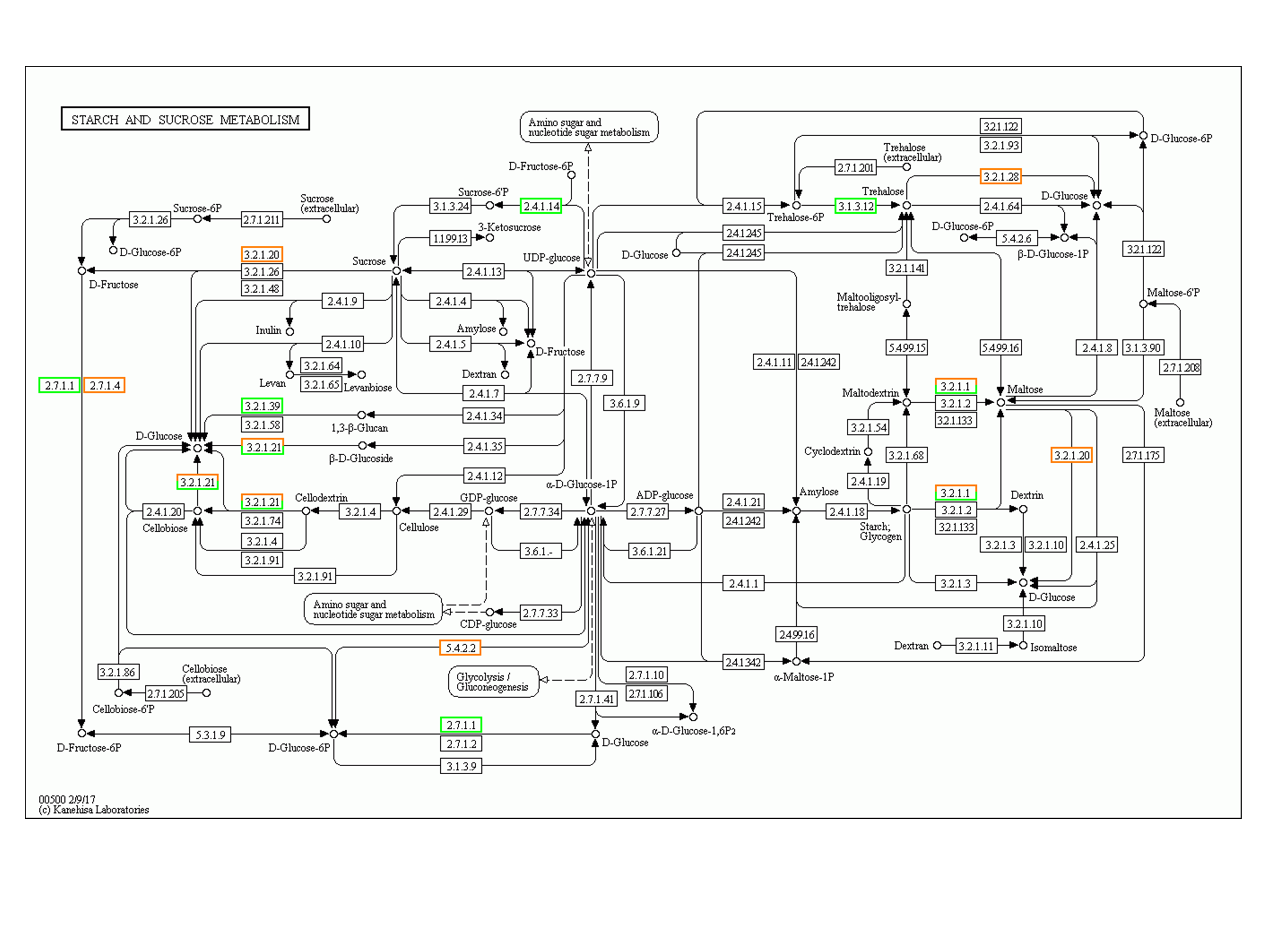

Supplement: Supplementary file 3 — Supplementary file3 Suppl. Fig. S3 Differential expression genes in starch and sucrose metabolism pathway between WT and CsERF2-overexpressing transgenic N. tabacum (TIF 1972 kb) [file 425_2020_3426_MOESM3_ESM.tif]

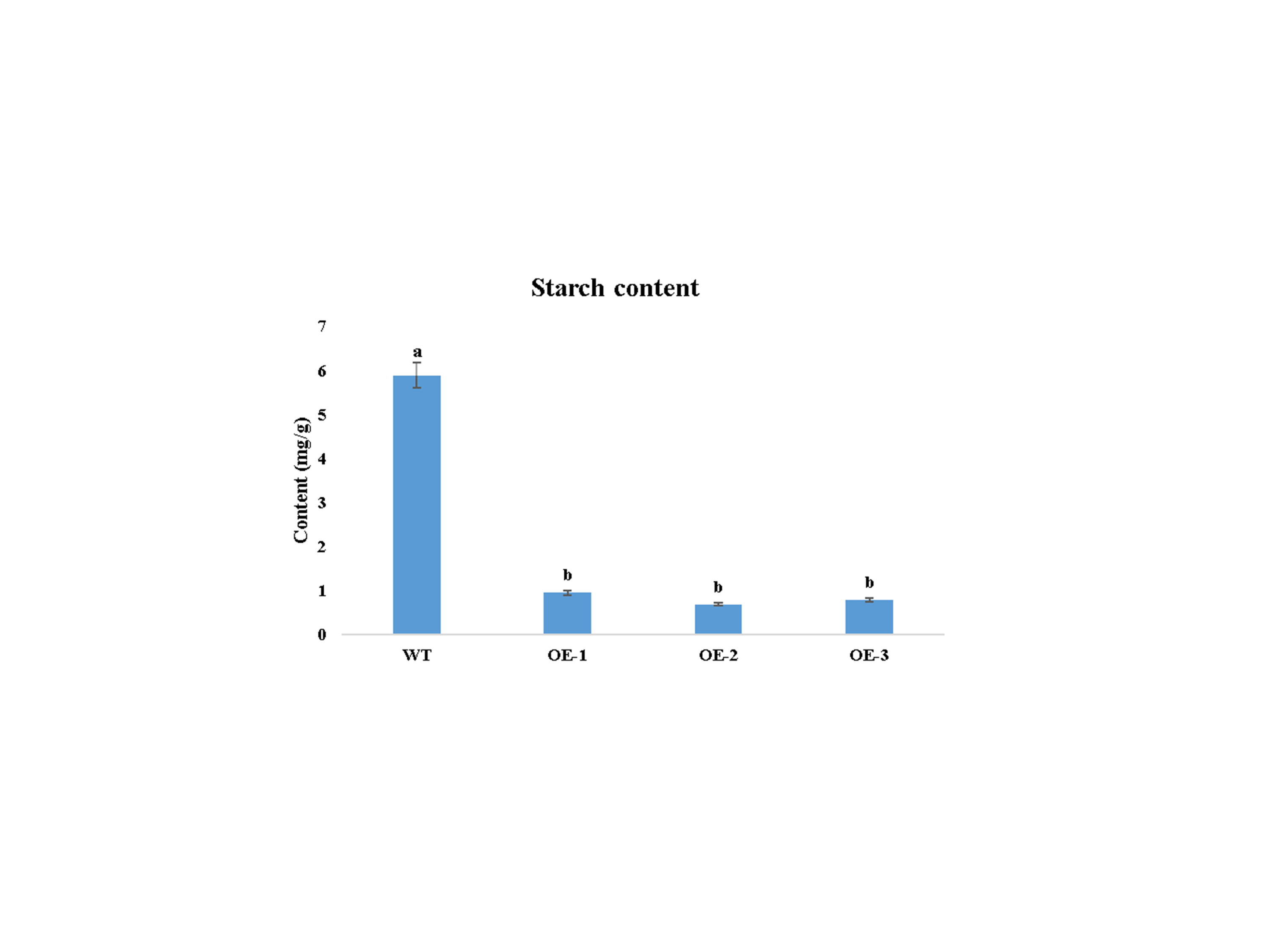

Supplement: Supplementary file 4 — Supplementary file4 Suppl. Fig. S4 Starch content in WT and transgenic N. tabacum plants. Data represent the means ± SE of at least three replicates. Different letters are used to indicate the means that differ significantly (P<0.05) (TIF 445 kb) [file 425_2020_3426_MOESM4_ESM.tif]

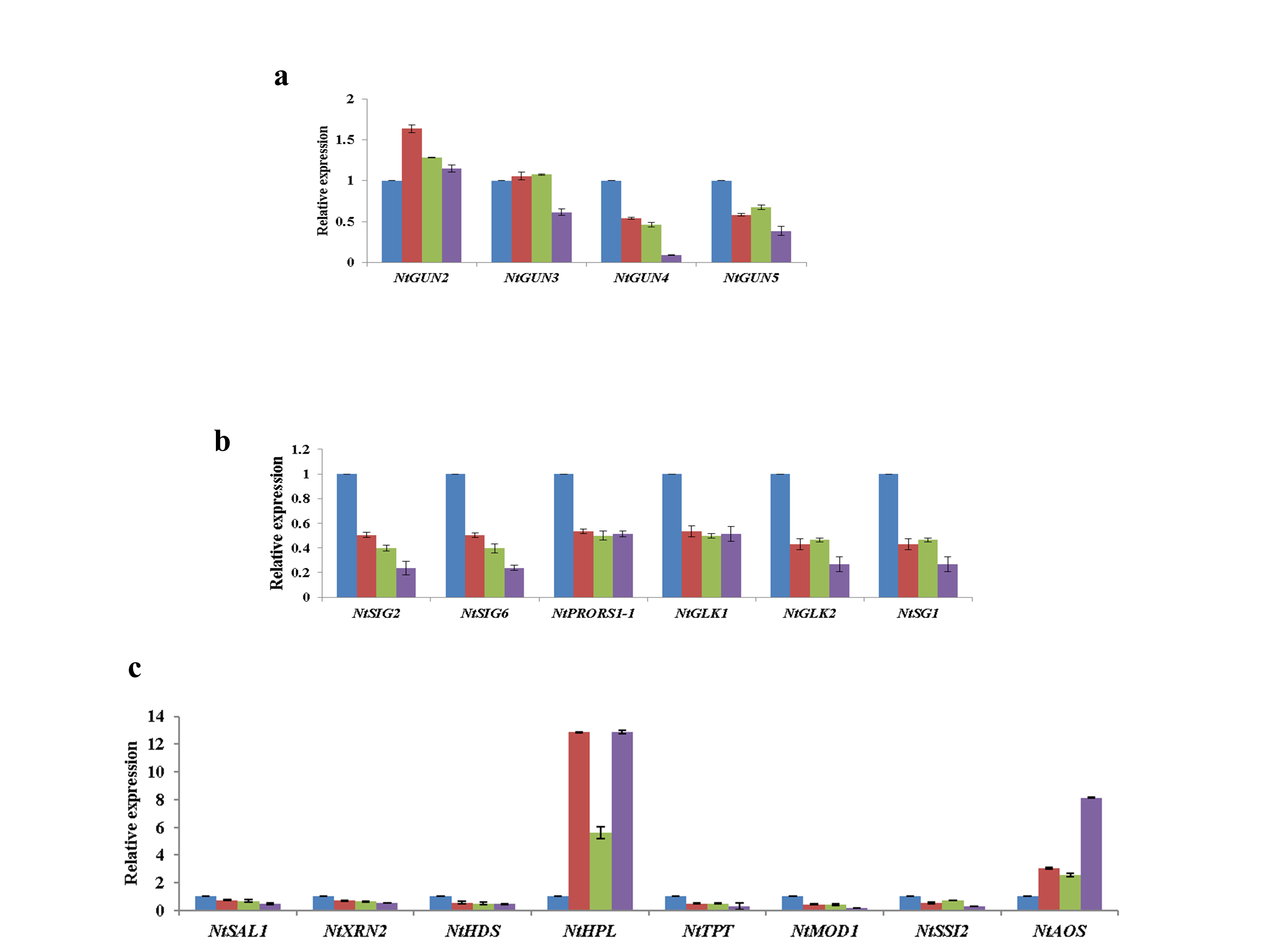

Supplement: Supplementary file 5 — Supplementary file5 Suppl. Fig. S5 Expression of genes a tetrapyrrole metabolism, b plastid gene expression signaling pathway, c plastid metabolism in transgenic N. tabacum. Relative expression was calibrated with WT. Data represent the means ± SE of at least three replicates (TIF 747 kb) [file 425_2020_3426_MOESM5_ESM.tif]
