# Supplementary material for: Involvement of CsERF2 in leaf variegation of Cymbidium sinense ‘Dharma’
Source: Planta. 2020 Jul 28;252(2):29. doi: 10.1007/s00425-020-03426-x (PMC7387381; doi:10.1007/s00425-020-03426-x)
Supplement: Supplementary file 8 — Supplementary file8 (DOCX 16 kb) [file 425_2020_3426_MOESM8_ESM.docx]

**Supplemental Table 3. Primers used for gene cloning and qPCR**

| **Genes** | **Forward primer (5'to 3')** | **Forward primer (5'to 3')** |
| --- | --- | --- |
| **Cloning** |  |  |
| *CsERF1* | ATGGCCAACTCCGCTCCTCCATC | TTACTCGTCGAACTTCCATAAAT |
| *CsERF2* | ATGGCCGATGCAGCCGAAACC | TTAGAAGTTCCAGAGAGATGA |
| *CsERF3* | ATGAACAGACCCGACGAGCTC | TCATACTGAGTGGCTCCACAG |
| *CsERF4* | ATGGACTCCTCCTCGGGCTCC | TCAGTAACTCCACAGAGAAAA |
| *CsERF5* | ATGGGCGATCGCCGTCGTGCC | AGAATCTCTGGTATTTCGATTAG |
| *CsERF6* | ATGGCCCCCAGGGAAAAGGGA | TCAGTTGAACTCAGCCGGAGG |
| *CsERF7* | ATGATGGCAGTCGATACTCTT | TCAAAGGAGAGGCGGTGGAAGAT |
| *CsERF8* | ATGGCCAACTCCGCTCCTCCATC | TTACTCGTCGAACTTCCATAAAT |
| *CsERF9* | ATGGCCGACCCCACTCCGACT | CTACTCATCATCGAACTTCCA |
| *CsERF10* | ATGGTGCCCAATCAGCAACCC | TCATCGTCGGGTATCATTCGG |
| **qPCR** |  |  |
| *CsERF1* | TCCCTACAAAACCCCGAAGA | AAGTGCCGAGCCAGATGC |
| *CsERF2* | GCAGACGATGTGCCAATG | GCTCCAAATGTTCTTCCTGAT |
| *CsERF3* | ATGAACAGACCCGACGAGC | GGAGACGGACGCCCTTATAG |
| *CsERF4* | CTCAGACGAGGGCTCCTACA | CAGCGGACGCCTTTGTAGAT |
| *CsERF5* | CGGTGACTCCAACTCCAAG | CTAATCGAAATACCAGAGATTCT |
| *CsERF6* | GTTCAGAGGGTCAAAGGCGA | GGAGGAATAGGGGTTGCGAG |
| *CsERF7* | GCGAGGAGAAGTCGGTTTCA | CCCAGCCATTTCCTCGTCTT |
| *CsERF8* | TCCCTACAAAACCCCGAAGA | AAGTGCCGAGCCAGATGC |
| *CsERF9* | CCACTCCGACTCTCCACAAG | TAGGTTCCAAGCCAGATGCG |
| *CsERF10* | CAAGTGGTTGGGGACATTC | CGTCGGGTATCATTCGGT |
| *CsCLH* | GCACGCAGTGAAGCTCCTTA | GGAAATTCCCCTTCCTCGCA |
| CsPPH | AAGCCATCCAAGCTCTCTCG | TGATGTGATCTCCTCGGGCT |
| CsPAO | TCGAAGCCGTTCTCTCAACC | AAGAAGCGGAAGAAGTCGCT |
| CsRCCR | CATCGATCTCGTTCCTCGCA | CTCGAGGTCTCTCCTTGGGA |
| CsACTIN | CAATGAGCTTCGTGTTGCCC | GATACGAACCAGTTGTGCGG |
| NtTUBA1 | CAACCTCTGTGGTGGAACCT | GTGTATGTGGGTCGCTCAAT |
| NtGUN2 | TTCCTGAGTATGCTGAGT | CATAACCTTGCTGCCTAA |
| NtGUN3 | CGAGTCTCTGAGATTCTT | GCCTTGTAATAATCCTTGAA |
| NtGUN4 | CGTTAGAGAATACCAGAA | CGTTAGAGAATACCAGAA |
| NtGUN5 | AGTGCCAGTTAGTTATGT | GTATGATGATTGTTAGTTCCT |
| NtSIG2 | TGTTGTCTCTGTGAAGTC | TCAAGTAACGCAATGGAT |
| NtSIG6 | GATAAGGCAAGGAGTTAG | TGGTTGGATGATGATTAC |
| NtPRORS1-1 | TGATGTGTCTTCATATAATG | TTACCTTCAATTCTTCCT |
| NtGLK1 | ATCCTCCTCATCTTCATC | ATATCTCGTTGGTGCTAT |
| NtGLK2 | CAGTAGAGCAATTAGGTGTAG | ATGTTATGGCGAGTGAGA |
| NtSG1 | TCTTGGTTCATCCTTCTT | GTCTTAGTAGCAGTTGTTG |
| NtAPX2 | AAGTGAGCGAAGAGTATGAA | GGAGGATAATAGGAGCACAA |
| NtSAL1 | CTCTGGAGACCTTCGTAA | AGCAAGCACATCTTCTTC |
| NtXRN2 | GTCTGTTGTTGAGGTTAA | GATTCCGTTCATATCCAA |
| NtXRN3 | ACCTTCTCCAACAACATT | AACACCATCAATAGCCATA |
| NtXRN4 | TTCTCATTAGTCCGTCCAA | GCTGCTGGTTCATCTTAG |
| NtHDS | CTTCCACCAGTAGATGATAA | CGACAGAGGAGTAATGAC |
| NtHPL | TACTGGAGATATGCGTGTT | GCTCATTGACAAGTGTAGG |
| NtTPT | CCTGCCATTATTATTGAG | CTGATTGTAGAGGTGATA |
| NtTRX-f1 | GATTGCTCCAAAGTTTCA | TAACGACCTTATTATTCTTCAG |
| NtZDS | TGGTAGACTATCTTGGTAA | CTTATCTCCTTAACATTCCT |
| NtMOD1 | GGCAATATCAGCATCAAGT | CTCAGACGCAATATAGGTAAG |
| NtSSI2 | ATGAACAAGTCAAGGAAT | CCAGTGTATTAAGCATAGT |
| NtAOS | GCGAACTTCAACAATATCA | GTCCATTAGAGCCAAGAT |
